# The Agency of Forced Migrants: Historical Perspectives

**DOI:** 10.1177/00220094251401610

**Published:** 2025-12-16

**Authors:** Philipp Strobl, Christoph Rass, Kerstin von Lingen

**Affiliations:** 27258 Department of Contemporary History, University of Vienna, Vienna, Austria; Institut für Migrationsforschung und Interkulturelle Studien, University of Osnabrück, Osnabrück, Germany; 27258 Department of Contemporary History, University of Vienna, Vienna, Austria

**Keywords:** forced migration, refugee agency, migration regimes, displaced persons

## Abstract

This introduction sets out the thematic and methodological framework of a special issue on historical refugee agency in the aftermath of the Second World War. It situates the volume within historiographical debates that move beyond narratives of passive victimization, instead foregrounding how refugees actively shaped their lives and the regimes that sought to govern them. The central question is how forced migrants exercised personal, proxy, and collective agency within severe constraints, and how such practices were connected across geographical and institutional contexts. Methodologically, the introduction highlights strategies for recovering refugee voices from fragmentary sources: reading bureaucratic records against the grain, tracing transnational networks, and identifying how administrative silences can register agency. It also underscores the value of the migration regime framework, intersectionality, and everyday practices in connecting perspectives on structural conditions with those on individual and collective action . In outlining these foundations, the introduction positions the special issue as a historiographical intervention that rethinks refugee history through the lens of agency.

The Second World War triggered one of the largest displacement crises in human history. Amid this upheaval, refugees exercised agency in multiple forms – negotiation of bureaucracies, forging of networks, and influencing of emerging migration regimes – demonstrating that the human capacity to act persists even under extreme constraint. How did they do so? How did they shape the very systems designed to control them? This special issue examines refugee agency in the aftermath of The Second World War, when millions of displaced people confronted emerging international migration regimes. Through case studies from Europe, Asia, and South America, we demonstrate that refugees were not passive recipients of humanitarian assistance but active participants who influenced policy implementation and institutional development in many different ways.

The Second World War's forced migration crisis presented unprecedented challenges to existing frameworks for understanding human mobility.^1^ The forced mobility generated by The Second World War affected a wide range of populations, including Holocaust survivors, forced labourers, political refugees, and war-displaced civilians across multiple continents. In response, Allied authorities introduced the administrative category of ‘displaced person’ (DP) to classify and manage millions of individuals who were either preparing to return to their pre-war homes or could not – or would not – return to their countries of origin. Other displaced populations, such as German-speaking expellees from Eastern Europe, who were associated with former enemy states, however, were not encompassed by this category. Rather than serving merely as bureaucratic convenience, the DP classification became a locus of negotiation between state power, international organizations, communities, and individuals.^2^

The contributors to this volume show that those labelled as DPs, as well as other postwar refugees, actively challenged and transformed the emerging postwar migration regime through various approaches. These ranged from refusing Soviet repatriation in European camps to mobilizing patriotic discourse in wartime China and creating transnational networks to negotiate competing bureaucracies. By deploying what we define as personal, proxy, and collective forms of agency, forced migrants influenced how international organizations such as the United Nations Relief and Rehabilitation Administration (UNRRA), the Chinese National Relief and Rehabilitation Administration (CNRRA) and the International Refugee Organization (IRO) conceptualized and implemented their operations.^3^ Examining these activities across geographical and institutional contexts reveals how displaced people became active participants in shaping systems designed to control and assist them, thereby leaving lasting imprints on the national and international structures of refugee governance. Each article of this special issue revisits aspects of the twentieth century's largest displacement crisis, namely violence-induced mobility caused by the Second World War and international efforts to address this humanitarian catastrophe. Our contributors offer fresh perspectives on the capacities of refugees for action, informed by conceptual advances in migration studies. Drawing on social cognitive theory, especially sociologist Albert Bandura's framework, we understand agency as the capacity of individuals or groups to make choices and act in ways that affect their lives and trajectories.^4^ Following Bandura, we distinguish three modes: (1) direct personal agency: individual actions taken to influence one's immediate environment; (2) proxy agency: reliance on others who possess greater resources or access; and (3) collective agency: socially coordinated efforts that amplify individual and collective capacities.^5^ This framework underscores the notion that individuals are not passive recipients of external conditions but actively construct their circumstances through deliberate and purposive actions.^6^ We argue that the framework offers crucial insights into how forced migrants navigated the constraints of postwar displacement. William Sewell Jr. recently challenged conventional understanding by arguing that agency pervades human interaction as a constant feature of social life. Rather than identifying agency only in dramatic resistance, Sewell shows how people continuously modify their social structures through everyday practices. Understanding forced migrants requires recognizing how they exercised self-determination as an omnipresent aspect of human experience, transforming migration regimes through incremental variations that officials rarely recognized as agential.^7^

Our central question is as follows: how did forced migrants exercise personal, proxy, and collective agency within emerging postwar migration regimes, and how were these forms connected across geographical and institutional contexts? Through case studies spanning Europe, Asia, and South America, we investigate what their agency looked like. We will see, for example, how refugees negotiated bureaucracies, created networks, adapted identities, and influenced governing systems. Even under extreme limitations, refugees and DPs acted as negotiators, and their approaches and activities significantly influenced the development of postwar migration regimes. This special issue seeks to contribute to the ongoing discussion by connecting these diverse histories to uncover how forced migrant autonomy operated transnationally, revealing patterns of action that transcended specific contexts while remaining attentive to local variations and constraints.^8^

The displacement crisis triggered by The Second World War had distinct but interconnected patterns across different continents. In Europe, seven to eight million ‘DPs’ were scattered across Western occupation zones by 1945. These included 2.5 million Poles, 1.6 million Soviet citizens, 700,000 French, 600,000 Italians, approximately 250,000 Jewish survivors, 200,000 Balts, and significant numbers from virtually every other European nation.^9^ In Asia, the Sino–Japanese War displaced an estimated 95 million Chinese civilians, figures that dwarfed even Europe's staggering numbers.^10^ Millions more sought refuge across the Americas, Africa, and Oceania.^11^

These numbers, incredible as they are, represent individual human tragedies and triumphs that statistics simply cannot capture. Each figure encompasses countless personal histories of loss, survival, and reconstruction that shaped the postwar world. UNRRA coordinated immediate relief and repatriation, successfully returning approximately seven million DPs by 1947. Yet as the Cold War loomed, the ‘last million’ (including approximately 400,000 Poles, 200,000 Balts, 150,000 Ukrainians, and 100,000 Yugoslavs) refused repatriation, fearing persecution in Soviet-controlled territories.^12^ The IRO, established in 1946, shifted its focus towards resettlement, facilitating the relocation of over one million DPs between 1947 and 1952.^13^ However, these statistics only partially capture how forced migrants actively shaped the systems designed to manage them, from refusing Soviet repatriation to mobilizing networks and negotiating bureaucratic categories.

The CNRRA operated under conditions that were distinct from those of UNRRA, managing millions of internally DPs within a nation still experiencing civil war.^14^ While UNRRA primarily handled cross-border displacement requiring diplomatic negotiations, CNRRA faced the challenge of managing internal displacement amidst ongoing conflict. These operational differences created divergent opportunities and constraints for refugee autonomy, as Jiayi Tao explored in her contribution to this volume. These institutional differences mattered profoundly. They shaped not only what was possible but also how refugees understood and navigated their options.

Scholarship on the resettlement programme reflects broader historiographical shifts in understanding refugees’ capacities for action. Foundational studies by Wolfgang Jacobmeyer and Michael Marrus in the mid-1980s interpreted this migration regime as a Western instrument of power for stabilizing postwar Europe, treating refugees primarily as targets of state management. Three decades later, Philipp Ther's *Die Außenseiter* and Matthew Frank's examination of population transfers signalled new approaches, with Ther particularly recognizing how refugees actively influenced European integration rather than merely being subjected to it.^15^ The evolution from state-centred analysis to the recognition of refugee autonomy parallels wider transformations in migration historiography that have influenced all recent work in this field.

Gerard Cohen's later work marked a historiographical turning point by acknowledging DPs as actors within an emerging international order rather than passive objects of state policy.^16^ Recent research increasingly traces how DPs manoeuvred bureaucratic categories, mobilized collective resources, and influenced policy implementation through their everyday activities and organized resistance. In this context, the IRO came to be interpreted as laying the groundwork for today's global refugee regime, with scholars drawing connections from the IRO's introduction of individual case assessment to the 1951 Geneva Convention's principle of individual persecution as grounds for refugee status.^17^

Research increasingly focuses on autonomy of survivors and camp experiences. Holocaust historians have examined Jewish Shoah survivors from this perspective. Atina Grossmann's pioneering work on trauma, memory, and motherhood among Jewish DPs revealed gendered dimensions of survival and recovery, while Avinoam Patt and Michael Berkowitz illustrated survivors’ collective capacities through cultural production and political organizing in DP camps.^18^ Recent studies by Annika Heyen and Sebastian Musch have highlighted the impact of Jewish organizations on the emergence of the postwar and post-Holocaust migration regime.^19^ These studies transcend narratives of victimization by foregrounding the ways in which survivors reconstructed their lives and communities, thereby anticipating contemporary scholarly recognition of refugee agency. As Michel Agier has shown, refugee camps themselves became sites of negotiation and resistance, where forced migrants transformed spaces designed for control into arenas that asserted autonomy and dignity.^20^

Historical research has examined Polish, Ukrainian, and Baltic DP collective experiences, analysing their self-determination in European camps and German society during repatriation or resettlement processes. Anna Holian's comparative study shows how different national groups developed distinct approaches for avoiding repatriation and securing resettlement. Jan-Hinnerk Antons traces Ukrainian DP manoeuvring between nationalist politics and pragmatic adaptation, a delicate balance that required constant negotiation.^21^ Systematic analysis of refugee autonomy, however, remains limited, although recent collaborative research on DP experiences in Germany and Austria has begun to address this gap.^22^ The studies expose the varied approaches employed by different groups based on the specific circumstances and resources available to them.

While refugee experience descriptions are often framed by ‘loss’, Ismee Tames and Christoph Rass suggest understanding violence-induced migration beyond loss alone, as a process of reconstruction and forward movement that produces specific knowledge and experience useful to forced migrants through amplified capacities.^23^ Such a perspective shifts the focus from what refugees lost to what they created, built, and transformed through displacement. In not defining refugees in terms of deficits, this approach recognizes the productive capacities that emerge from experiences of displacement, a crucial reframing that influences how we interpret the cases in this volume.

The global scope of displacement extended well beyond Europe. Diana Lary's study of Chinese civilian experiences demonstrates how mass dislocation reconfigured Chinese society by fostering new social networks and political mobilizations. Similarly, Stephen MacKinnon's scholarship and Robert Capa's visual documentation of Wuhan highlight refugees not as passive casualties of Japanese aggression but as pivotal agents in wartime mobilization.^24^ The resettlement of DPs in Australia provides a particularly compelling lens on refugee agency. While the Australian postwar migration programme was structured around government priorities and labour needs, DPs actively navigated these frameworks, negotiating placement, employment, and social integration in ways that shaped both their own lives and broader settlement policies. Their agency is visible in the formation of community networks, cultural associations, and efforts at advocacy, which not only facilitated adaptation but also influenced public perceptions and policy development. These processes demonstrate that, even within highly structured resettlement schemes, DPs were not passive recipients of state decisions but proactive actors shaping postwar Australian society.^25^

Taken together, these geographically diverse cases underscore that postwar displacement was a worldwide phenomenon that demanded transnational responses while simultaneously generating new forms of migrant autonomy across borders.

Migration scholarship has undergone a significant transformation since the 1990s cultural turn, evolving from a peripheral concern to a central topic in historiography. Traditional structure-based explanations relied on simplistic push–pull models, quantitative analyses, and abstract neoclassical frameworks of supply and demand, portraying migration as determined by states, economic forces, or violence.

By the end of the twentieth century, migration scholars had increasingly adopted qualitative approaches that foregrounded the previously marginalized autonomy of migrants themselves. The ‘new economics of migration’ theory developed models explaining migration through individual and collective choices, while sociologists introduced the concept of the ‘autonomy of migration’.^26^ These approaches solidified the shift from deterministic models towards identifying dialectical relationships between migrant actions and structural constraints.^27^ Instead of viewing structure and agency as opposing forces, scholars have begun examining their mutual constitution through activities, including those of migrants, a perspective that informs all contributions to this special issue.

Across the articles in this issue, a variety of approaches have been employed to analyse refugee agency, and the following pages highlight the most significant of these perspectives. The transformation from viewing refugees as ‘speechless emissaries’ to recognizing their political agency fundamentally shapes our analysis. Liisa H. Malkki's 1996 critique of humanitarian practices that transform refugees into universal victims stripped of political identities resonates throughout this volume, especially in Kerstin von Lingen's examination of returnees whose complaint letters were dismissed as ‘ungrateful’ rather than acknowledged as legitimate political claims.^28^ Building on Malkki's insights, Bhupinder S. Chimni's challenge to Northern-produced narratives from a Global South perspective illuminates how the ‘myth of difference’ between past and present refugees justifies restrictive policies, a dynamic that Jiayi Tao's analysis of Chinese refugees directly confronts by showing continuities in refugee strategic adaptations across different contexts.^29^

Oliver Bakewell's 2008 call for ‘policy-irrelevant research’ that studies forced migrants’ actual activities rather than policy categories alone is essential for understanding the cases presented here.^30^ His framework helps us see how refugees constantly negotiate, subvert, and reconstruct management systems: this is precisely what Christoph Rass and Jessica Wehner uncover through their Muslim DP's strategic identity shifts, and what Philipp Strobl traces in Austrian *Volksdeutsche*'s Habsburg mythology creation. Also, Strobl's empirical findings support Nicholas de Genova's theoretical insight that migrants’ persistent movements constitute ‘tactics of bordering’ that challenge state sovereignty.^31^ Franziska Lamp's analysis of refugee camp workers, on the other hand, exemplifies Didier Fassin's observation of how refugees develop approaches to working through humanitarian bureaucracies while asserting dignity within constraints.^32^

The ‘autonomy of migration’ framework's emphasis on ‘imperceptible politics’, everyday activities that gradually transform borders and citizenship regimes from below, manifests concretely in our case studies.^33^ Polish displaced children exercised agency through acts misinterpreted as misbehaviour, as Samantha Knapton shows in her article. Annika Heyen's analysis of governmental inaction furthermore exposes the calculated strategy behind apparent paralysis. Both cases demonstrate how power operates through seemingly minor or negative actions. This dialectical understanding, as Sabine Hess argued regarding 2015's ‘summer of migration’, captures moments when autonomy of migrants becomes so visible that it forces recognition of the autonomous dimensions of human mobility, a phenomenon that parallels the postwar ‘last million’ DPs whose collective refusal of repatriation fundamentally altered international refugee governance.^34^ Recent scholarship on the temporal dimensions of protracted displacement enriches our understanding of the cases presented. Cathrine Brun's concept of ‘active waiting’ and Melanie Griffiths's ‘sticky time’ help us understand how refugees in our studies managed immobility while maintaining hope.^35^ Temporal dimensions of agency emerge across our contributions: in von Lingen's account of returnees who endured Canadian conditions while persistently pressing for repatriation, in Strobl's analysis of Volksdeutsche sustaining cultural narratives across generations, and in Knapton's study of children preserving Polish identity despite pressures of assimilation. The recent work of José Renkens and colleagues on ‘agency of inaction’ provides theoretical grounding for understanding Annika Heyen's Bermuda Conference analysis, where deliberate non-action constituted purposeful agency, with devastating consequences.^36^

While these theoretical frameworks provide valuable analytical tools, the historical reality of violence-induced mobility often exceeds or complicates neat theoretical categories and models. As several contributions to this volume show, refugee experiences sometimes defy our attempts at systematic theorization. The messiness of lived experience tends to resist overly rigid academic ordering.

The reflexive turn in migration research questions how scholars study and categorize forced migrants. Janine Dahinden argues that categories like ‘migrant’, ‘refugee’, or ‘migration background’ represent normative political constructs bound to nation-state logic and colonial history. When researchers unreflectively employ such categories, they inadvertently reproduce ‘migrantizing’ worldviews that reinforce racist and nationalist debates. This critique directly informs our contributors’ methodological approaches: each critically reinterprets bureaucratic categories, revealing how DPs actively traversed, manipulated, and subverted the classifications meant to turn them into a manageable population.^37^ Rogers Brubaker's distinction between analytical and practical categories underscores the ways in which migrants engage with, resist, and strategically appropriate bureaucratic classifications. The majority of our contributions reveal the resulting disjunctions between administrative taxonomies and lived experience.^38^ Several contributions in this volume draw on the migration regime framework developed by Frank Wolff and Christoph Rass, which provides an analytical lens for exploring how diverse actors (states, international organizations, NGOs, and migrants) interacted to shape mobility patterns, positioning migrants as active agents rather than passive subjects.^39^

Intersectionality is crucial for understanding the differentiated capacities of refugees in our case studies. The interplay of gender, race, class, and sexuality generated specific configurations that either enabled or constrained agency. This is evident in Erker's study of Nazi refugees, who leveraged class and racial privilege in Argentina; in Knapton's analysis of Polish children, who faced multiple vulnerabilities; and in Lamp's examination of the gendered dimensions of camp administrative roles.^40^ These intersecting identities directly shaped bureaucratic categorization during negotiations between institutions and individuals, with certain combinations conferring advantages and others generating multiple barriers. Crucially, these structural inequalities framed – but did not wholly determine – the possibilities for exercising self-determination.^41^

While violence-induced mobility during the war years has attracted scholarly attention to the capacities of victims, systematic analysis of how survivors exercised self-determination and agency in the postwar period remains underdeveloped, as the limited body of studies on historical refugee agency indicates.^42^ Little is known from a historical perspective about the practices and agency of forced migrants and the mediation and promotion of their knowledge. This source-related invisibility stems from the partial absence of archives dedicated to preserving migrant testimonies. Instead, forced migrants appear in historical records primarily through bureaucratic encounters, such as registration, relief distribution, medical examination, and resettlement screening, rather than through their own narratives.^43^

Despite these limitations, this special issue employs diverse methodological strategies to recover evidence of migrant self-determination. Contributors seek out scarce biographical traces of refugees, read administrative documents against the grain to identify moments of autonomy in bureaucratic encounters, and reconstruct networks from archival fragments that official records were never designed to preserve. Some analyses infer agency indirectly from institutional responses, reasoning that administrative adjustments often reflected refugee influence. Knapton's study of children, reconstructed through adult testimonies, illustrates how careful interpretation can recover voices from sources that otherwise suppress autonomy. Such approaches demand patience and creativity, but they yield crucial insights into how forced migrants understood and actively shaped their circumstances.

Biographical cases of transnational journeys help overcome methodological nationalism by examining trajectories across space, time, and varying contexts.^44^ This approach historicizes migrant autonomy by examining how individuals, within their capacities, negotiate societal opportunities and constraints in the pursuit of their life plans.^45^ In this context, agency serves as a lens for observing context-specific category production in migration regimes through negotiations between institutions and individuals, as demonstrated in this special issue among others by Rass and Wehner and Strobl.

What emerges from our special issue is not a uniform pattern of refugee agency but rather a complex tapestry of strategies, adaptations, and negotiations in the sense of a connected histories approach. The contributions examine various manifestations of historical refugee agency: decision-making regarding destinations and movement timing; resistance through cultural identity preservation and challenging discriminatory norms; adaptation processes through which migrants identified with new societies while maintaining origin connections; network creation for supporting new arrivals and transmitting crucial knowledge; and transnational capacities for maintaining connections to their homeland through remittances of money, political engagement, and cultural exchange. Following recent theoretical developments, we also recognize agency in calculated waiting, endurance, and deliberate non-action as forms that preserve energy and opportunities.^46^

Individual capacities for life control varied considerably according to opportunity structures such as educational background, language proficiency, social capital, and the ability to activate networks.^47^ While some refugees could draw on pre-existing connections, others were compelled to construct new structures from the ground up, as Strobl's case study of the collective agency of German-speaking refugees in postwar Austria illustrates. Across the contributions, qualitative perspectives are employed to analyse the agency–structure nexus, in line with a ‘history from below’ approach. Evidence is reconstructed from diverse sources: personal letters smuggled from camps despite censorship; oral histories that capture both memory and deliberate forgetting; photographs of daily life; crafts preserving cultural traditions; and camp newspapers through which refugees articulated their own narratives.

The diverse histories examined in this special issue reveal distinct yet interconnected patterns of refugee self-determination across different geographical regions in the aftermath of the Second World War. From European DPs refusing repatriation to Chinese refugees mobilizing patriotic discourse, from Jewish survivors organizing camp life to Nazi refugees relocating to South America, forced migrants demonstrated remarkable capacities to navigate and reshape their disrupted lives. As the contributions show – often in unexpected ways – refugees not only shaped their own circumstances but also influenced the very systems designed to govern them. The following case studies illustrate how forced migrants exercised personal, proxy, and collective forms of agency, frequently in overlapping and simultaneous ways. They are organized into three analytical categories: (1) Navigating Bureaucratic Classifications Through Strategic Self-Representation, (2) Intermediary Roles and Networks, and (3) Agency at Both the Centre and the Margins.

Four contributions are located in category one. They examine how refugees strategically manipulated their identities within bureaucratic systems, revealing that categorization became a site of negotiation rather than a simple imposition. Christoph Rass and Jessica Wehner present a meticulous analysis of a refugee categorized as ‘Muslim’ who adapted identity multiple times within postwar Germany's DP system. Through careful reconstruction of administrative correspondence, they trace how this individual acquired a deep understanding of bureaucratic systems and international politics, using this knowledge to shift between categories depending on which status offered access to mobility or immobility at specific moments.^48^ The case reveals sophisticated calculations about when to emphasize religious identity, when to claim national belonging, and when to invoke humanitarian protection. Chinese refugees during wartime displacement, as Tao demonstrates, developed parallel strategies in a vastly different context. Her analysis of non-elite refugees reveals how they presented themselves as ‘patriots’ while, as she pointedly notes, ‘caring much less about China's political future than their own destiny’. This appropriation of nationalist discourse for pragmatic ends shows how refugees creatively reinterpreted state narratives to serve their personal needs. The strategic manipulation of identity was not limited to individual cases. In postwar Austria, German-speaking refugee organizations exposed collective construction of identity on a remarkable scale, as Strobl reveals. The Zentralberatungsstelle für Volksdeutsche created elaborate Habsburg heritage myths, deliberately presenting themselves as ‘Old Austrians’ to distance themselves from Nazi Germany associations, but also from other refugees. This collective myth-making involved coordinated construction of narratives that influenced Austria's integration policies – a striking example of how shared storytelling became a tool for political positioning, although the effectiveness of these narratives varied considerably depending on the audience. Kerstin von Lingen takes a different approach by examining ‘returnees’, a term referring to resettled DPs who rejected placements and demanded repatriation to Europe. Her critical interpretation of IRO archives reveals exercises of resistance in correspondence dismissed as ‘ungrateful.’ Cases like Alessandro M., who denounced the ‘mille promesse’ that never materialized, forced the IRO to confront its regime's limits. What the organization termed ‘failure’ represented successful resistance to exploitative conditions. These returnees transformed apparent defeat into strategic victory through persistent letter-writing, hunger strikes, and work refusal, although not all such resistance proved successful, and many returnees found themselves trapped between impossible choices. Together, these four cases demonstrate how forced migrants across vastly different contexts developed sophisticated understandings of bureaucratic expectations and deliberately performed identities that maximized their opportunities, although success was never guaranteed, and many attempts at strategic self-representation failed or backfired.

The two contributions that form our second category explore how refugees gained agency through strategic positioning within institutional structures under dramatically different circumstances. Through refugee camp administration employment, Franziska Lamp explores the intersection of proxy and personal agency. Her research brings to light how UNRRA depended entirely on DP employees for translation, cultural mediation, and practical administration, a dependency that created unexpected opportunities for refugee agency, though one that could also lead to exploitation.^49^ These refugee workers occupied a crucial intermediary position, mediating between bureaucratic requirements and community needs. They often bent rules to assist compatriots while maintaining privileged positions that enabled resource accumulation essential for resettlement. Their structural positioning allowed them to simultaneously serve as representatives of the UNRRA authority to fellow DPs and as advocates for interests of refugees within the administration. This dual role created unique opportunities for exercising both personal and proxy agency. Linda Erker's examination of German and Austrian National Socialist refugees in Argentina presents a more troubling case of institutional positioning. Unlike other ‘DPs’, who faced scrutiny and restrictions, Nazi refugees leveraged established networks with German-Argentine business communities, Catholic Church connections, and sympathetic elements within the Perón government. They successfully avoided the stigma of ‘undesired’ refugee categorization while securing privileged positions in Argentine society. Pre-existing diaspora networks, professional skills, and ideological sympathies provided them with forms of autonomy unavailable to most forced migrants. This case starkly illustrates how pre-existing resources and social capital fundamentally shaped displacement experiences, raising uncomfortable questions about who benefits from institutional networks.

The final pair of contributions, belonging to our third category, address agency exercised under the most extreme constraints by those typically considered either utterly powerless or overwhelmingly powerful. Samantha Knapton approaches displaced Polish children's autonomy through innovative methodology, analysing welfare worker and military testimonies to access seemingly voiceless subjects. Her careful analysis exposes remarkable patterns of resistance. Unaccompanied children resisted categorization through activities consistently misinterpreted as misbehaviour: escaping from camps to search for family members; refusing adoption to preserve Polish identity; maintaining cultural practices despite intense assimilation pressure; and creating informal networks for protection and information sharing. Even the most vulnerable forced migrants asserted preferences that forced the constant adaptation of child welfare policies. The self-determination of these children manifested through acts that adults systematically failed to recognize as deliberate choices, exposing profound biases in how we understand and document agency. Annika Heyen's analysis presents the inverse case: governmental agency exercised through calculated inaction during the 1943 Bermuda Conference.^50^ Here, the most powerful actors, Allied governments, employed the rhetoric of the ‘war effort’ as a sophisticated screen to avoid assisting Jewish refugees while appearing responsive to public pressure. Through a meticulous analysis of diplomatic correspondence, Heyen shows how officials deliberately structured discussions to reach predetermined conclusions. They limited agendas, excluded Jewish organizations from meaningful participation, and choreographed proceedings to ensure that no substantial commitments emerged. This ‘organized hypocrisy’ reveals how state agency exercised through calculated non-action affected possibilities for refugees as powerfully as any positive intervention. The conference's carefully engineered status quo deflected criticism while condemning countless refugees to death. These contrasting cases (vulnerable children versus powerful governments) reveal that agency operates across all levels of power, manifesting through both action and deliberate inaction. They remind us that power and powerlessness are never absolute.

This special issue demonstrates that mid-twentieth-century forced migrants actively shaped both their own circumstances and the systems designed to regulate them. Through personal, proxy, and collective agency, DPs transformed postwar migration regimes from below, thereby challenging conventional understandings of refugee history. At the same time, it is essential to recognize that such agency was exercised within severe constraints, and many efforts at self-determination ultimately remained unsuccessful.

Recent theoretical advances demand a critical examination of how we study forced migration. Janine Dahinden's warning about unreflective use of state-produced categories must inform how we read sources and interpret refugee actions.^51^ The ‘autonomy of migration’ approach reveals how migrant movements continuously exceed and transform control systems. This process was evident in our case studies.^52^ William Sewell Jr.'s insight that agency pervades all social interaction is important here. Even under extreme constraints, displaced people constantly deployed their capacities through both action and calculated inaction.^53^

Future research on historical refugee agency could pursue several promising directions. Comparative studies across displacement contexts can illuminate how agency manifests under different structural conditions. Longitudinal research tracking how capacities evolve through displacement and resettlement phases would deepen our understanding of temporal dimensions. We need to examine cases of unsuccessful exercise of autonomy to better understand constraints and limitations. An analysis of transmission of knowledge within displaced communities could reveal how strategies and tactics circulated across networks.^54^ These historical patterns offer essential insights into contemporary displacement crises, reminding us that today's refugees, like their historical predecessors, are never passive victims.

Through careful empirical work guided by reflexive theoretical frameworks, historians can contribute to a more comprehensive understanding of forced migration. Such scholarship enhances not only our knowledge of the past but also our capacity to analyse present challenges and anticipate future developments in refugee studies. The stories told here remind us that recognizing refugee agency is not merely an academic exercise, it is essential for understanding how human beings navigate and transform even the most constraining circumstances.

## Notes

1 F. Nuscheler, *Internationale Migration: Flucht und Asyl* (Wiesbaden 2015).

2 S. Huhn and C. Rass, ‘Displaced person(s): The production of a powerful political category’, *Ethnic and Racial Studies*, 48, 4 (2024), 718–39, DOI: 10.1080/01419870.2024.2404488.

3 L. Holborn, *The International Refugee Organization: A Specialized Agency of the United Nations. Its History and Work* (London 1956).

4 A. Bandura, ‘Social cognitive theory: An agentic perspective’, *Annual Review of Psychology*, 52 (2001), 1–26.

5 Bandura, ‘Social Cognitive Theory’, 2.

6 A. Bandura, ‘Toward a psychology of human agency’, *Perspectives on Psychological Science*, 1, 2 (June 2006), 164–80.

7 W.H. Sewell Jr., ‘Is agency a useful historical concept?’, *Journal of Social History*, 57, 3 (2024), 436–40, DOI: 10.1093/jsh/shad063.

8 C. Rass, ‘International migration’, in D. Schlosser, B. Badie and L. Morlino (eds) *Handbook of Political Science* (London 2020), 1474–89.

9 W. Jacobmeyer, *Vom Zwangsarbeiter zum Heimatlosen Ausländer: Die Displaced Persons in Westdeutschland 1945–1951* (Göttingen 1985); G. Cohen, *In War*'*s Wake: Europe's Displaced Persons in the Postwar Order* (New York 2011); J. Oltmer, *Migration: Geschichte und Zukunft der Gegenwart* (Darmstadt 2017), 145.

10 Oltmer, *Migration: Geschichte und Zukunft der Gegenwart*, 145; R.K. Schoppa, *In a Sea of Bitterness: Refugees during the Sino-Japanese War* (Cambridge, MA 2011).

11 S. Korbel and P. Strobl, *Cultural Translation and Knowledge Transfer on Alternative Routes of Escape from Nazi Terror: Mediations through Migrations* (London 2021).

12 M. Wyman, *DP: Europe*'*s Displaced Persons, 1945–1951* (Philadelphia, PA 1989).

13 Holborn, *The International Refugee Organization*.

14 R. Mitter, ‘Imperialism, transnationalism and the reconstruction of postwar China, 1944-6’, *Past and Present*, 218, (2013), 51-69; J. Tao, ‘Towards development: The Yellow River project and UNRRA's technical assistance to China, 1944–1947’, *Journal of Global History*, 19, no. 2 (2024), 301–21. https://doi.org/10.1017/S1740022824000032.

15 M. Marrus, *The Unwanted: European Refugees in the Twentieth Century* (New York 1985); P. Ther, *Die Außenseiter: Flucht, Flüchtlinge und Integration im modernen Europa* (Berlin 2017); M. Frank, *Making Minorities History: Population Transfer in Twentieth-Century Europe* (Oxford 2017).

16 Cohen, *In War*'*s Wake*.

17 G. Loescher, *The UNHCR and World Politics: A Perilous Path* (Oxford 2001).

18 A. Grossmann, ‘Trauma, memory, and motherhood: Germans and Jewish displaced persons in Post-Nazi Germany, 1945–1949’, *Archiv für Sozialgeschichte*, 38 (1998), 215–39; A. Patt and M. Berkowitz, *‘We are Here’: New Approaches to Jewish Displaced Persons in Postwar Germany* (Detroit 2010).

19 A. Heyen and S. Musch, ‘The Bermuda Conference in April 1943: Allied politics, Jewish organizations and the emergence of the International Migration Regime’, *Holocaust Studies*, 30, 2 (2024), 290–307.

20 M. Agier, *Managing the Undesirables: Refugee Camps and Humanitarian Government* (Cambridge 2011); N. Sigona, ‘The politics of refugee voices: Representations, narratives, and memories’, in E. Fiddian-Qasmiyeh, G. Loescher, K. Long and N. Sigona (eds) *The Oxford Handbook of Refugee and Forced Migration Studies* (Oxford 2014), 369–82.

21 J.-H. Antons, *Ukrainische Displaced Persons in der britischen Zone: Lagerleben zwischen nationaler Fixierung und pragmatischen Zukunftsentwürfen* (Essen 2014); A. Holian, *Between National Socialism and Soviet Communism: Displaced Persons in Postwar Germany* (Ann Arbor, MI 2011).

22 N. Hagen et al. (eds), *Displaced Persons-Forschung in Deutschland und Österreich: Eine Bestandsaufnahme zu Beginn des 21.* *Jahrhunderts* (Berlin 2021).

23 C. Rass and I. Tames, ‘Negotiating the aftermath of forced migration: A view from the intersection of war and migration studies in the digital age’, *Historical Social Research*, 45, 4 (2020), 7–44; Strobl pursues a similar approach: P. Strobl, *A History of Displaced Knowledge: Austrian Refugees from National Socialism in Australia* (Leiden 2025).

24 D. Lary, *The Chinese People at War: Human Suffering and Social Transformation, 1937–1945* (Cambridge 2010); S. MacKinnon and R. Capa, *Wuhan, 1938: War, Refugees, and the Making of Modern China* (Berkeley, CA 2008).

25 S. Fitzpatrick, *Lost Souls: Soviet Displaced Persons and the Birth of the Cold War* (Princeton 2024); R. Balint, *Destination Elsewhere: Displaced Persons and their Quest to Leave Postwar Europe* (Ithaca 2021), J. Persian, *Fascists in Exile: Post-War Displaced Persons in Australia* (London 2023).

26 S. Hess, ‘Border crossing as act of resistance: The autonomy of migration as theoretical intervention into border studies’, in M. Butler, P. Mecheril and L. Brenningmeyer (eds) *Resistance: Subjects, Representations, Contexts* (Bielefeld 2017), 87–100.

27 O. Bakewell, ‘Some reflections on structure and agency in migration theory’, *Journal of Ethnic and Migration Studies*, 36, 10 (2010), 1689–708.

28 L. Malkki, ‘Speechless emissaries: Refugees, humanitarianism, and dehistoricization’, *Cultural Anthropology*, 11, 3 (1996), 377–404.

29 B. Chimni, ‘The geopolitics of refugee studies: A view from the south’, *Journal of Refugee Studies*, 11, 4 (1998), 350–74.

30 O. Bakewell, ‘Research beyond the categories: The importance of policy irrelevant research into forced migration’, *Journal of Refugee Studies*, 21, 4 (2008), 432–53.

31 N. de Genova, *The Borders of ‘Europe’: Autonomy of Migration, Tactics of Bordering* (Durham, NC 2017).

32 D. Fassin, *Humanitarian Reason: A Moral History of the Present* (Berkeley, CA 2011); J. Hyndman, *Managing Displacement: Refugees and the Politics of Humanitarianism* (Minneapolis, MN 2000).

33 D. Papadopoulos, N. Stephenson and V. Tsianos, *Escape Routes: Control and Subversion in the Twenty-First Century* (London 2008); G. Castillo Ramírez, ‘Autonomy of Migration: From the Political Production of Borders to Migrant Struggles’, *Migraciones Internacionales*, 14, Artículo 07 (2023), 1–25.

34 Hess, ‘Border Crossing as Act of Resistance’.

35 C. Brun, ‘Active waiting and changing hopes: Toward a time perspective on protracted displacement’, *Social Analysis*, 59, 1 (2015), 19–37; M. Griffiths, ‘Out of time: The temporal uncertainties of refused asylum seekers and immigration detainees’, *Journal of Ethnic and Migration Studies*, 40, 12 (2014), 1991–2009, DOI: 10.1080/1369183X.2014.907737; M. Emirbayer and A. Mische, ‘What is agency?’, *American Journal of Sociology*, 103 (1998), 962–1023.

36 J. Renkens, E. Rommes and M. van den Muijsenbergh, ‘Refugees’ agency: On resistance, resilience, and resources’, *International Journal of Environmental Research and Public Health*, 19, 2 (2022), 806.

37 J. Dahinden, ‘…When the category “migration” lost its innocence for migration scholars. And what now? A plea for dialogue’, *Comparative Migration Studies*, 13, Art. 37 (2025), DOI: 10.1186/s40878-025-00459-7.

38 R. Brubaker, ‘Categories of analysis and categories of practice: A note on the study of Muslims in European countries of immigration’, *Ethnic and Racial Studies*, 36, 1 (2013), 1–8; S. Coutin, *Legalizing Moves: Salvadoran Immigrants**’ Struggle for US Residency* (Ann Arbor 2000).

39 A. Pott, C. Rass and F. Wolff, *Was ist ein Migrationsregime? What Is a Migration Regime?* (Wiesbaden 2018).

40 N. Yuval-Davis, ‘Power, intersectionality and the politics of belonging’, in W. Harcourt (ed) *The Palgrave Handbook of Gender and Development* (London 2016), 367–81; E. Kofman and P. Raghuram, *Gendered Migrations and Global Social Reproduction* (London 2015).

41 H. Crawley and D. Skleparis, ‘Refugees, migrants, neither, both: Categorical fetishism and the politics of bounding in Europe's “migration crisis”’, *Journal of Ethnic and Migration Studies*, 44, 1 (2017), 48–64; L. Borelli et al., ‘Agency within mobility: Conceptualising the geopolitics of migration management’, *Geopolitics*, 27, 4 (2022), 1140–67.

42 P. Strobl, ‘Collective refugee agency and the negotiation of migration laws in wartime Australia, 1939–1943,’ *The Historical Journal*, 68 (2025), 627-46; W.H. Sewell Jr, ‘Is agency a useful historical concept?’, *Journal of Social History*, 57, 3 (2024), 436–40.

43 M.-R. Trouillot, *Silencing the Past: Power and the Production of History* (Boston 1995).

44 B. Lüthi, ‘Migration and Migration History’, Version: 2.0, Docupedia-Zeitgeschichte, 2018. Available at: http://docupedia.de/zg/Luethi_migration_v2_en_2018 (accessed 6 August 2025).

45 C. Harzig, D. Hoerder and D. Gabaccia, *What is Migration History?* (Cambridge 2009).

46 Renkens, Rommes and van den Muijsenbergh, ‘Refugees’ Agency’.

47 P. Strobl, ‘Social networks of Austrian refugee-migrants from the Anschluss in Australia – An analysis of meaning structures’, *Journal of Migration History*, 5, 1 (2019), 53–79.

48 Coutin, *Legalizing Moves*.

49 See Hyndman, *Managing Displacement*.

50 Heyen's article, initially intended for inclusion in this special issue, was published in volume 61(1). Heyen, A. (2026). ‘A Façade for Inaction’? Governmental Actors, the Bermuda Conference and the Non-Rescue of Jewish Refugees. Journal of Contemporary History, 61(1), 28–48. https://doi.org/10.1177/00220094251375957.

51 Dahinden, ‘…When the Category “Migration” Lost its Innocence’.

52 Castillo Ramírez, ‘Autonomy of Migration’; Borelli et al., ‘Agency within Mobility’.

53 Sewell Jr., ‘Is Agency a Useful Historical Concept?’, 436.

54 Strobl, *A History of Displaced Knowledge*.

